# Case Report: ABO-incompatible living donor liver transplantation in a patient with associated Rosai–Dorfman–Destombes disease, first reported case

**DOI:** 10.3389/frtra.2025.1576301

**Published:** 2025-04-24

**Authors:** Bhabani Sankar Sahoo, Kausar Makki, Vikas Saini, Piyush Srivastava, Vivek Vij

**Affiliations:** Department of Liver Transplantation and Surgical Gastroenterology, Fortis Hospital, Noida, India

**Keywords:** RDD, Rosai–Dorfman–Destombes disease, ABOi, ABO-incompatible, LDLT and DDLT, chronic liver disease (CLD)

## Abstract

Rosai–Dorfman–Destombes disease (RDD), a rare histiocytic proliferation, is often associated with lymphadenopathy and extranodal manifestations, including involvement of the liver. We report a unique case of RDD presenting with chronic liver disease (CLD) in a 7-year-old boy, highlighting the association between these conditions. The patient underwent ABO-incompatible living donor liver transplantation (LDLT), a procedure not previously documented in the context of RDD. Successful transplantation was preceded by a desensitization protocol including rituximab and immunoadsorption, and was followed by a satisfactory postoperative course. This case underscores the need for further investigation into the relationship between RDD and CLD and the potential of LDLT as a life-saving treatment option in such complex cases.

## Introduction

Rosai–Dorfman–Destombes disease (RDD) is a rare clinicopathological entity characterized by histiocytic proliferation, clinically presenting as bilateral cervical lymphadenopathy, often accompanied by fevers, night sweats, and weight loss (1, 2). Its prevalence is approximately 1 in 200,000, with the condition most frequently diagnosed in children and young adults, averaging around 20 years of age (3, 4). The etiology of RDD remains unclear (5, 6). Classic nodal involvement occurs in up to 60% of cases, while approximately 40% may present with extranodal manifestations affecting the skin and soft tissue, central nervous system, eyes and orbits, and upper respiratory tract. Rarely, the urogenital (4%) and gastrointestinal systems (<1%) can be involved (7). We report a unique case of RDD associated with chronic liver disease (CLD), successfully treated with ABO-incompatible (ABOi) living donor liver transplantation (LDLT), a scenario not previously documented, although an association between RDD and CLD has been reported (8). Informed consent was provided by the patient’s parents for this submission.

## Presentation of case

**Figure 1:**
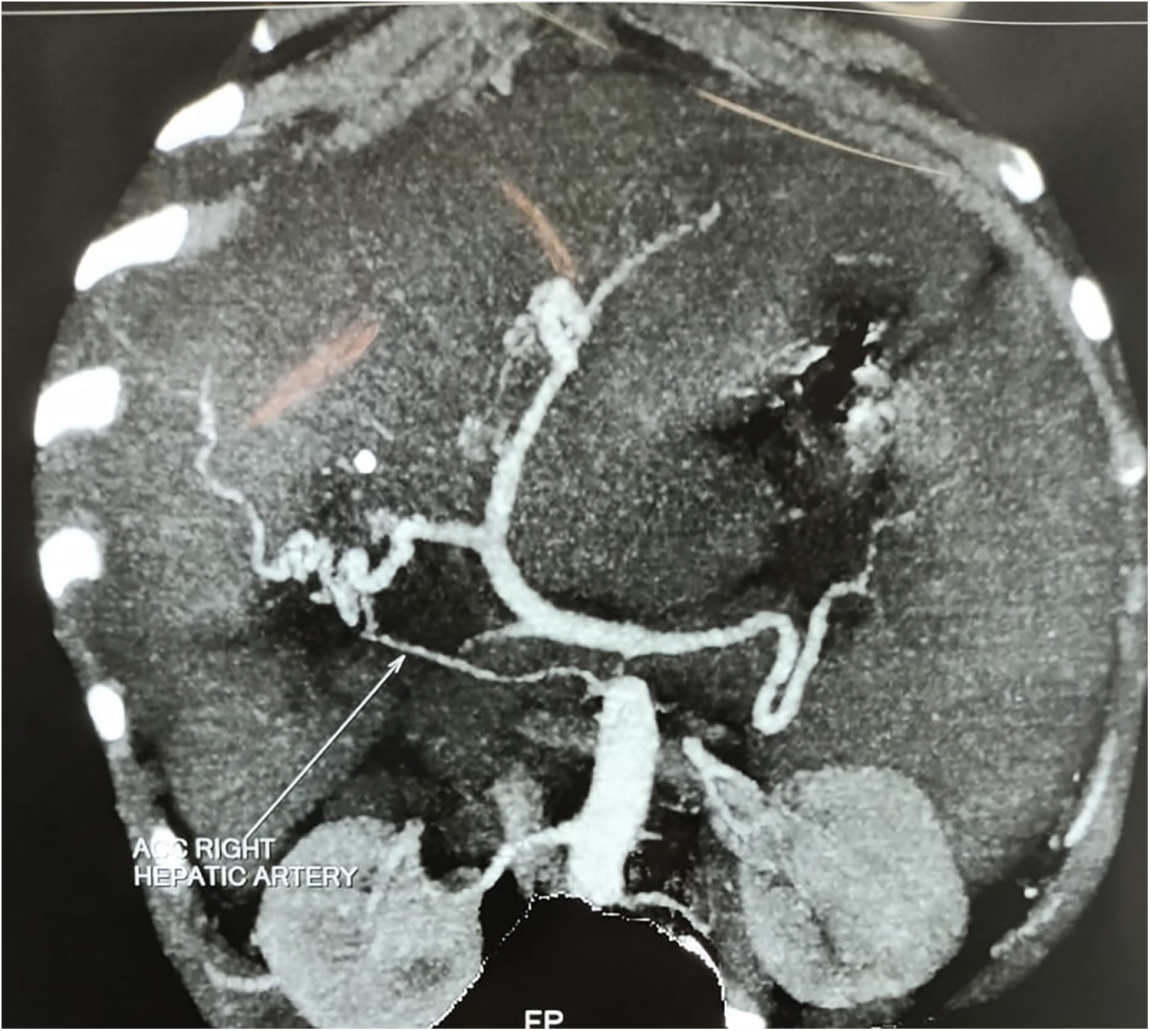
CT scan showing hepatomegaly and splenomegaly.

A 7-year-old boy from Tanzania presented with jaundice, abdominal distension, and severe anemia. He had undergone cholecystectomy at age 5, followed by two re-explorations within a month, likely for a bile leak. He was sarcopenic and his weight was 22 kg. Abdominal examination revealed hepatosplenomegaly. Routine blood investigations are shown in Table 1. An ultrasound of the abdomen revealed hepatosplenomegaly, ascites needing multiple taping, and jaundice. There was no history of variceal bleeding, hepatic encephalopathy, or renal impairment. As part of the diagnostic protocol, a triphasic computed tomography (CT) scan of the abdomen and chest was performed, showing enlarged liver and spleen (Figure 1), multiple enlarged periportal and retroperitoneal lymph nodes, and ascites. The chest CT scan further revealed enlarged mediastinal lymph nodes and bilateral cervical lymph nodes, cardiomegaly, and pericardial effusion. A positron emission tomography (PET) scan, conducted due to the generalized lymphadenopathy, demonstrated multiple mildly ^18^F-fluorodeoxyglucose (FDG)-avid lymph nodes in the left supraclavicular fossa, thorax, abdomen, and retroperitoneum. Left supraclavicular lymph node excision biopsy and ultrasound-guided true-cut liver biopsy were performed. The lymph node biopsy confirmed RDD, while the liver biopsy showed only features of cirrhosis. Immunohistochemistry (IHC) used for diagnosing RDD in the lymph node was negative in the liver biopsy. The etiology of CLD in this case was not ascertained after a thorough workup. The family was counseled on the need for LDLT because of the decompensated CLD (Child C), and ABOi LDLT was planned, as no suitable donor was found with a compatible blood type (recipient: O positive, donor: A positive). We explained the advantages and disadvantages of ABOi LDLT to the patient’s family and also that there was a scarcity of literature regarding liver transplantation in the setting of RDD. Informed consent was obtained from his parents before the procedure. Following optimization and the necessary workup for LDLT, the patient received 300 mg of rituximab. Pre-rituximab, his anti-A titer was IgG/IgM 1:64/1:32. Immunoadsorption was performed according to the protocol 1 day prior to transplantation, yielding anti-A titers of 1:32/1:32 on the day of the transplant. The left lobe LDLT was successfully performed without significant intraoperative events. Postoperatively, the patient was placed on a triple immunosuppression regimen [steroids, tacrolimus, and mycophenolate mofetil (MMF)]. His hospital stay was uneventful, and he was discharged on day 15 post-surgery. In the explant liver biopsy, we did not find any evidence of RDD, as there was no histiocytic and plasma cell infiltration, but there was evidence of cirrhosis-like distorted lobular architecture and bridging fibrosis.

**Table 1:** Relevant preoperative laboratory values.

Laboratory values				
CBC	HB-4.1 (g/dl)	TLC-7210	PLT-74000	
LFT	TB/DB-4.16/2.38 (mg/dl)	AST/ALT-52/10 (IU/L)	SAP/GGT-618/182 (IU/L)	PROTEIN/ALBUMIN-14/1.9 (g/dl)
PT/INR	19/1.66			
RFT	BUN/CREATININE- 06/0.54 (mg/dl)	Na/K-129/3.58 (meq/L)		

CBC, complete blood count; LFT, liver function test; PT, prothrombin time; INR, International normalised ratio; RFT, renal function test; HB, hemoglobin; TB/DB, total and direct bilirubin; BUN, blood urea nitrogen; TLC, total leucocyte count; AST, aspartate aminotransferase; ALT, alanine transferase; Na, sodium; K, potassium; PLT, platelet; SAP, serum alkaline phosphatase; GGT, gama-glutamyl transferase.

## Discussion

**Figure 2:**
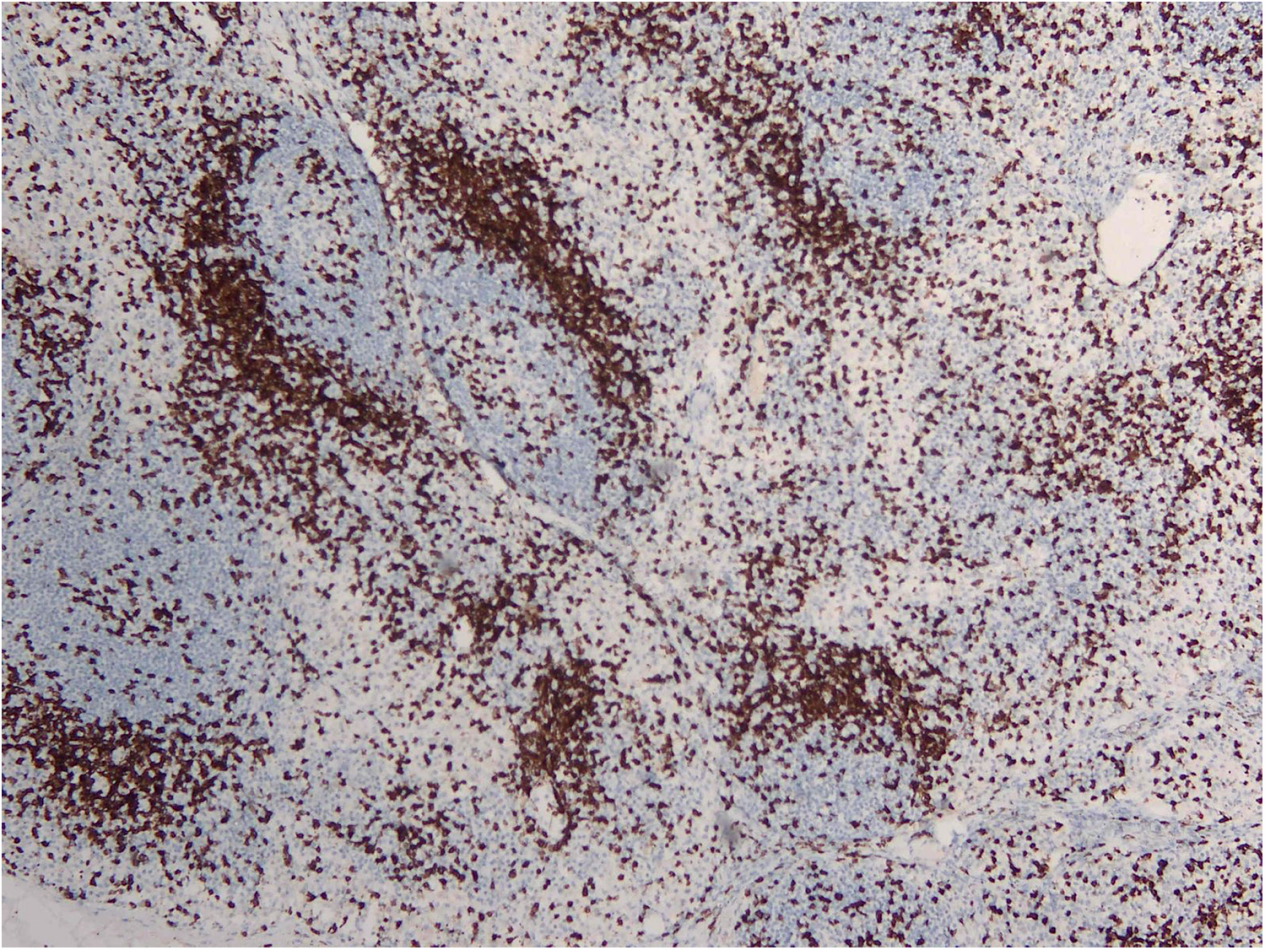
CD3 IHC stains showing a reactive T cell population.

**Figure 3:**
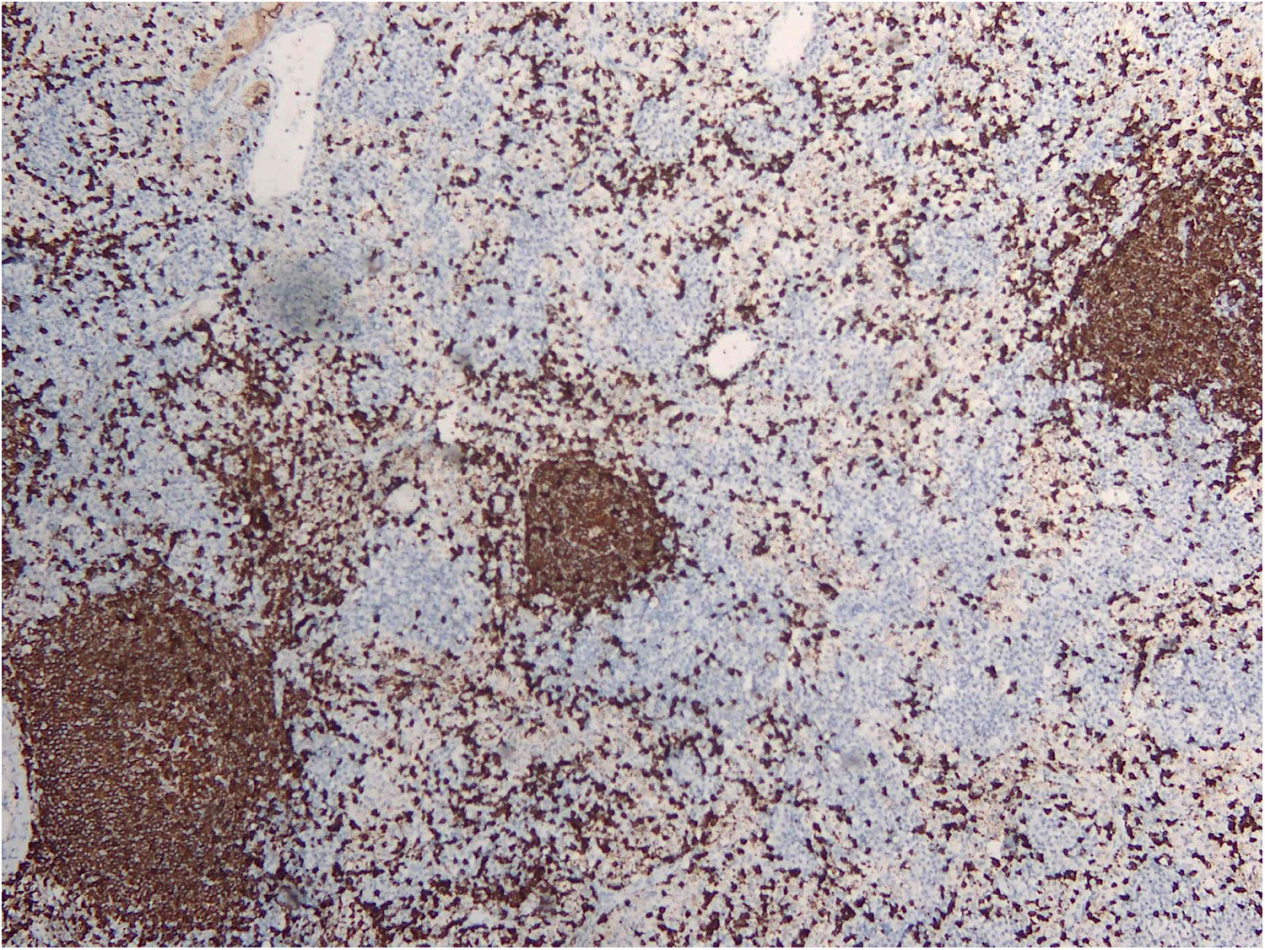
CD20 IHC showing a reactive B cell population.

**Figure 4:**
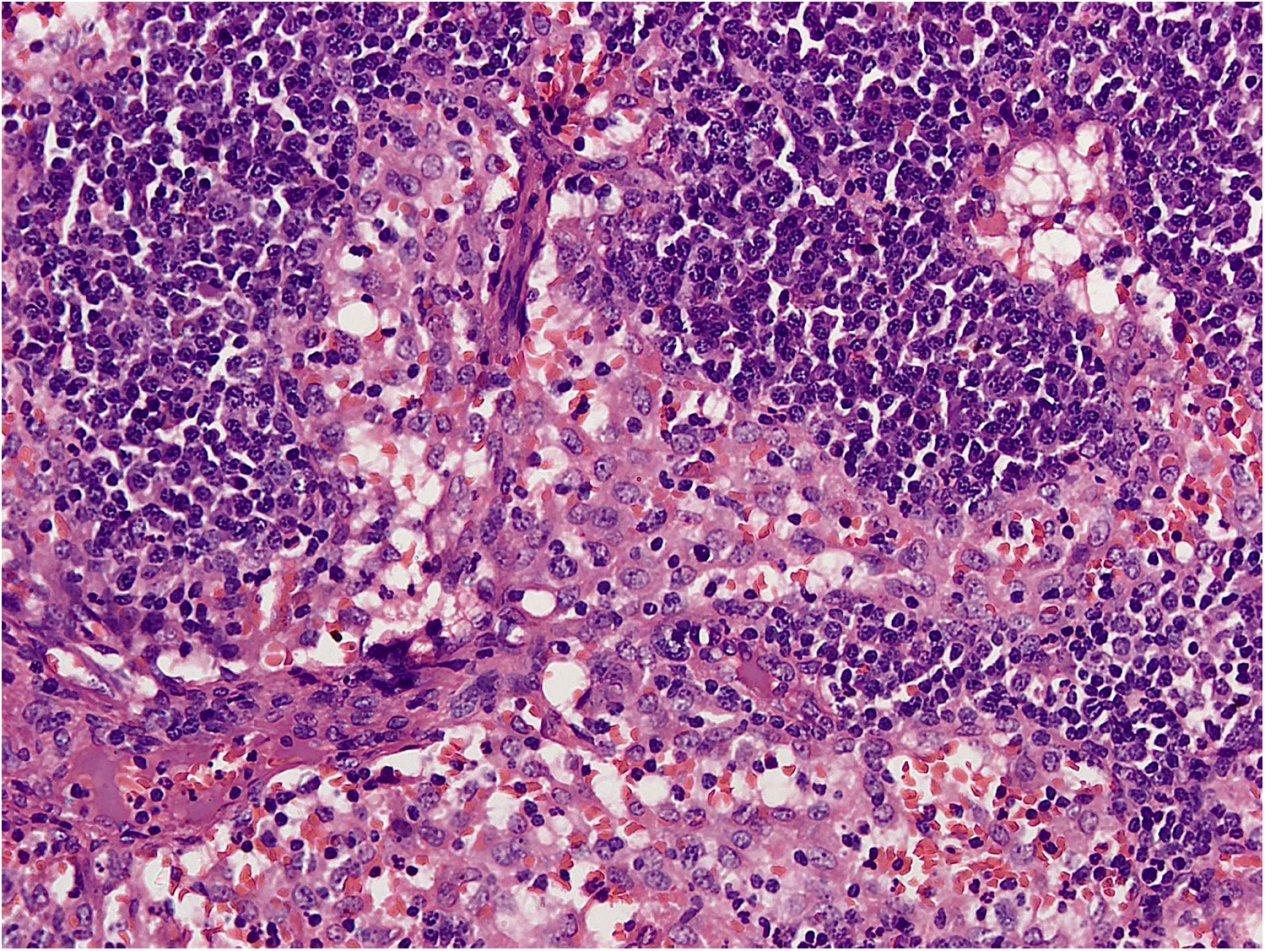
H&E section shows effaced nodal architecture. The sinuses are distended and filled with histiocytes.

RDD is a rare disease defined by emperipolesis, which refers to the infiltration and movement of lymphocytes into histiocytes, alongside significant sinusoidal dilation in affected lymph nodes. IHC results are typically positive for S100, negative for CD1a, and positive for CD3, CD4, and CD20 (Figures 2–4). While RDD can affect individuals of any age, it is most commonly diagnosed in the first and second decades of life, with a slight male predominance. Extranodal involvement is less common; however, the nodal variant often involves lymph nodes in the head and neck. Asymptomatic cases generally do not require treatment, and various options are available for symptomatic nodal disease, though these are not yet standardized (7).

In our case, a 7-year-old boy presented with features of CLD and was diagnosed with RDD upon evaluation. The association of RDD with CLD has not been previously documented. Sasaki et al. reported a case of RDD in a 55-year-old woman presenting with CLD and renal failure, where a liver biopsy indicated established cirrhosis, perisinusoidal fibrosis, and mild inflammation, predominantly with small lymphocytes and a few plasma cells. IHC was not performed on the liver biopsy in that case. In our case, the child had undergone open cholecystectomy at the age of 5. Gallbladder issues requiring cholecystectomy at such a young age are exceedingly rare. Arabadzhieva et al. presented a case of isolated gallbladder involvement in extranodal RDD presenting as cholecystitis, which may explain the cholecystectomy in our patient, despite their patient being 58 years old (8–10).

Regardless of the cause of CLD in this case, transplantation was necessary as a life-saving intervention. Due to the unavailability of compatible blood groups, an ABOi LDLT was planned. To date, liver transplantation in the context of RDD has not been documented in the literature. As part of the desensitization protocol for ABOi LDLT, the child received an injection of rituximab (300 mg) 2 weeks prior to the transplant. Rituximab has been suggested as a treatment for RDD; for example, Alqanatish et al. reported administering two doses of rituximab (500 mg/m^2^) 2 weeks apart to a child. Thus, rituximab proved beneficial in this case (11).

Several other treatment options for RDD, including steroids, methotrexate, cladribine, radiotherapy, imatinib, and thalidomide, have been described (7). In the postoperative period, the patient was managed with triple immunosuppression using steroids, tacrolimus, and MMF. A repeat PET scan at 3 months post-transplant showed a significant reduction in lymphadenopathy. A short follow-up period was the main limitation of this study. We did not ascertain the etiology of CLD in this patient and the association between RDD and CLD was also not established.

## Conclusion

The relationship between RDD and CLD remains unclear—whether RDD is a causal factor for CLD or merely an associated condition has yet to be established. Notably, LDLT in the context of RDD has not been previously reported in the literature. To the best of our knowledge, this represents the first documented case of LDLT for CLD in a patient with RDD.

## Data Availability

The original contributions presented in the study are included in the article/Supplementary Material, further inquiries can be directed to the corresponding authors.
